# Biochemical Prediction of Acute Cholangitis and Symptomatic Bile Duct Stones by Gallstone Hepatitis

**DOI:** 10.1155/1995/51915

**Published:** 1995

**Authors:** M. Isogai, K. Hachisuka, A. Yamaguchi, A. Hori

**Affiliations:** Department of Surgery Ogaki Municipal Hospital 4–86, Minaminokawa Ogaki 503 Japan

## Abstract

We have adopted the clinical concept of gallstone hepatitis indicated by marked serum transaminase elevation due to an acute inflammatory liver cell necrosis in the early stages of gallstone impaction in the bile duct as clinical and biochemical criteria for identifying high-risk patients for acute cholangitis or bile duct stones causing symptoms (symptomatic bile duct stones, SBDS).

One hundred and fifty-eight (80.2%) of 197 patients with acute gallstone disease and concomitant elevation of serum transaminase (gallstone hepatitis) underwent emergency treatment, either surgery (138 patients) or percutaneous transhepatic biliary drainage (PTBD)/endoscopic sphincterotomy (ES) (20 patients). One hundred and forty-two (89.9%) and 67 (42.4%) were confirmed to have SBDS and acute cholangitis, respectively, in the early stage of the disease. The majority of the patients who had no bile duct stones identified at surgery had either biliary pancreatitis or multiple small stones in the gallbladder. They were assumed to have migrating stones or false negative operative cholangiograms.

In conclusion, gallstone hepatitis indicates that SBDS and acute cholangitis are probable, and facilitates rapid selection of patients for urgent biliary tract exploration in patients with acute gallstone disease.


*HPB Surgery*, 1995, Vol. 8, pp. 267–273
Reprints available directly from the publisher
Photocopying permitted by license only

© 1995 OPA (Overseas Publishers Association)
Amsterdam B.V. Published in The Netherlands
by Harwood Academic Publishers GmbH
Printed in Malaysia

### Biochemical Prediction of Acute Cholangitis and Symptomatic Bile Duct Stones by Gallstone Hepatitis

M. ISOGAI, K. HACHISUKA, A. YAMAGUCHI and A. HORI

Department of Surgery, Ogaki Municipal Hospital, 4–86, Minaminokawa, Ogaki, 503, Japan

*(Received October 28, 1993)*

We have adopted the clinical concept of gallstone hepatitis indicated by marked serum transaminase elevation due to an acute inflammatory liver cell necrosis in the early stages of gallstone impaction in the bile duct as clinical and biochemical criteria for identifying high-risk patients for acute cholangitis or bile duct stones causing symptoms (symptomatic bile duct stones, SBDS).

One hundred and fifty-eight (80.2%) of 197 patients with acute gallstone disease and concomitant elevation of serum transaminase (gallstone hepatitis) underwent emergency treatment, either surgery (138 patients) or percutaneous transhepatic biliary drainage (PTBD)/endoscopic sphincterotomy (ES) (20 patients). One hundred and forty-two (89.9%) and 67 (42.4%) were confirmed to have SBDS and acute cholangitis, respectively, in the early stage of the disease. The majority of the patients who had no bile duct stones identified at surgery had either biliary pancreatitis or multiple small stones in the gallbladder. They were assumed to have migrating stones or false negative operative cholangiograms.

In conclusion, gallstone hepatitis indicates that SBDS and acute cholangitis are probable, and facilitates rapid selection of patients for urgent biliary tract exploration in patients with acute gallstone disease.

KEY WORDS: Gallstone hepatitis   acute cholangitis   symptomatic bile duct stones   gallstone pancreatitis   laparoscopic cholecystectomy

### INTRODUCTION

Acute cholangitis is a potentially life-threatening disease that results from biliary tract obstruction and subsequent infection. Recognition of the clinical manifestations is important in diagnosing and managing these patients[1]. However, reliance on Charcot's triad (abdominal pain, fever, and jaundice) is insufficient[1] and the clinical presentation can be nonspecific[2]. Accordingly, a diagnosis of acute cholangitis should be considered whenever there are signs of biliary tract obstruction and infection[1]. Ideally, bile duct stones causing symptoms (symptomatic bile duct stones, SBDS) should be identified early before acute cholangitis develops. With the rapid and widespread acceptance of laparoscopic cholecystectomy as the preferred primary operation for cholelithiasis, the identification of SBDS also becomes clinically important. What, then, is the diagnostic feature that identifies SBDS or biliary tract obstruction?

We have been involved in a clinicopathological study to clarify the cause of markedly elevated serum transaminases in patients with acute gallstone disease, which might have led to the diagnosis of so-called hepatitis. We concluded that serum transaminases might be raised markedly in the very early stage of biliary obstruction, that this highly elevated serum transaminases might be a reflection of an acute in-

Address for correspondence to: Masatoshi Isogai, M. D., Department of Surgery, Ogaki Municipal Hospital, 4-86, Minaminokawa, Ogaki, 503, Japan.

flammatory liver cell necrosis caused by impacted bile duct stones (gallstone hepatitis[3,4]), and that therefore gallstone hepatitis might be a sign of early biliary tract obstruction caused by impacted bile duct stones before serum bilirubin and alkaline phosphatase became elevated[4]. A case presented below provided further insight into the pathogenesis of gallstone hepatitis which could develop into severe acute cholangitis unless biliary decompression occurred[3].

Based upon this background and knowledge, we have adopted the clinical concept of gallstone hepatitis as a criterion for identifying patients with acute cholangitis or SBDS. At our hospital, almost all patients with acute gallstone disease who do not respond to initial conservative treatment are operated on immediately, after necessary biliary tract examination, provided the patients present a reasonable surgical risk. Here, we present a review of 197 patients who had gallstone hepatitis and therefore were suspected of having acute cholangitis or SBDS and were treated at the Department of Surgery, Ogaki Municipal Hospital, during the past 5 years. The main purpose of this study is to report the actual biliary pathology ultimately confirmed at surgery, especially in the acute stage of the disease, of patients with gallstone hepatitis.

### CASE STUDY

A 69-year-old female was referred to the Department of Gastroenterology, Ogaki Municipal Hospital, with epigastric pain and a liver function disorder which had developed two days before. Serum liver chemistry test results one day after the onset of symptoms were: glutamic oxalacetic transaminase (GOT) 2,380 (Karmen unit, normal <40); glutamic pyruvic transaminase (GPT) 747 (Karmen unit, normal <35); and bilirubin 2.0 (mg/dl, normal <1.2). She was diagnosed as having so-called hepatitis and underwent medical treatment. However, the abdominal pain did not abate and serum transaminases, especially GPT, remained high while serum bilirubin fluctuated (Table 1). On the sixth day after the onset of symptoms she developed a high fever and shock. An abdominal ultrasonography (US) scan on that day revealed gallstones in the dilated bile duct. The diagnosis of gallstone hepatitis followed by severe acute cholangitis was made, and she underwent percutaneous transhepatic biliary drainage (PTBD) (Fig. 1). Following this procedure, a rapid fall in serum transaminase and bilirubin activity and an immediate remission of symptoms were observed. After the resolution of cholangitis, she underwent delayed biliary surgery safely.

**Table 1** Time course of symptoms and liver chemistry

| *Day** | *Symptoms* | *GOT 2** | *GPT 3** | *T. bil4** |
|---|---|---|---|---|
| 0 | severe epigastric pain | ? | ? | ? |
| 1 | relief of pain | 2380 | 747 | 2.0 |
| 2 | slight pain | 788 | 863 | 6.3 |
| 4 | slight pain | 145 | 391 | 1.9 |
| 6 | high fever & shock | 175 | 368 | 14.0 |

* : Time from onset of symptoms
2* : Glutamic oxalacetic transaminase (Karmen unit, normal <40)
3* : Glutamic pyruvic transaminase (Karmen unit, normal <35)
4* : Total bilirubin (mg/dl, normal <1.2)

In this patient, the initial very high levels of serum transaminases (gallstone hepatitis) led to misdiagnosis of so-called hepatitis, and this led to a delay in biliary decompression and to the resultant development of severe acute cholangitis.

### PATIENTS AND METHODS

We have previously classified gallstone hepatitis as a new clinical entity[3,4]. The clinical diagnostic criteria for gallstone hepatitis are: (1) severe abdominal pain; (2) markedly elevated serum transaminase levels which might lead to the diagnosis of so-called hepatitis; and (3) gallstones, usually revealed in the dilated biliary tract, including the gallbladder, by using US. The histological features of gallstone hepatitis included: (1) degeneration and necrosis of liver cells (an accumulation of neutrophils in an area where liver cells had vanished from liver cell plates); and (2) acute cholangitis (neutrophil infiltration around and into the lumen of the bile duct in the portal triad). With regard to serum transaminase levels, the initial levels might depend on the extent of the liver cell necrosis caused by the impacted bile duct stones. The highly elevated serum transaminase levels during the onset of symptoms fall rapidly after resolution of the bile duct obstruction[3,4]. Serum transaminases, however, remain slightly elevated to various levels above normal when bile duct stones remain floating, causing bile statis. Thus, serum transaminase levels might depend on the extent of ductal obstruction or on the interval between the onset of the symptoms and the time of carrying out the biochemical tests. We believe, therefore, that serum transaminase elevation, no matter how slight, in patients with acute gallstone disease should lead doctors to suspect that patients have had gallstone hepatitis in the course of the disease.

Based upon the features of the course of serum transaminases in gallstone hepatitis, all patients with

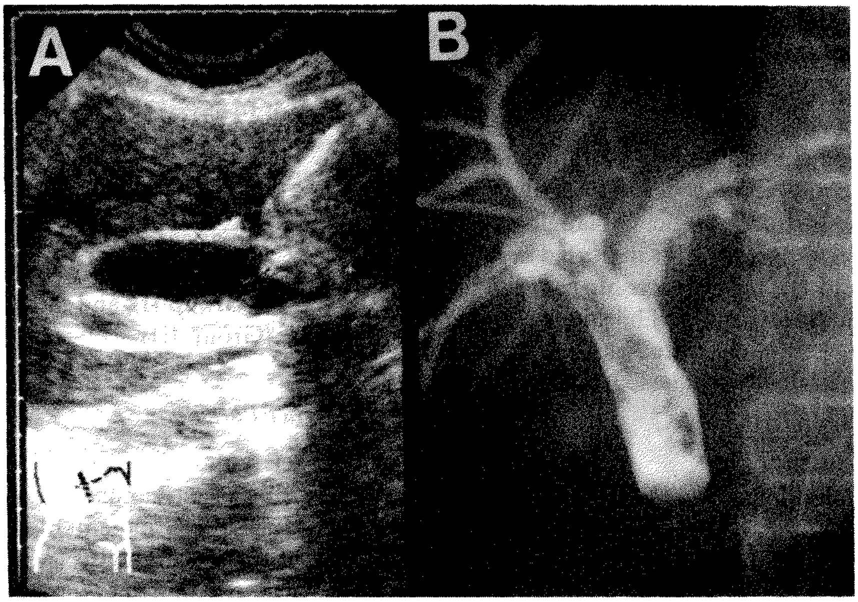

**Figure 1** Abdominal ultrasonography (US) on the sixth day after onset of symptoms revealed gallstones in the dilated bile duct (A). Percutaneous transhepatic cholangiography (PTC) showed complete obstruction of the bile duct (B).

gallstones and with serum transaminases at above-normal levels were diagnosed as having gallstone hepatitis. As a result, 197 patients were diagnosed as having gallstone hepatitis from January 1987 to December 1991. They were treated under suspicion of having acute cholangitis or SBDS, and entered into this study. Patients with viral hepatitis or other potential causes of transaminase elevation such as heart failure or drug or alcohol abuse were excluded. The patients were analyzed with respect to clinical presentation, preoperative examinations, management, biliary tract pathology, operative procedures, and outcome.

### RESULTS

### *Clinical Presentation*

There were 99 males and 98 females; age range was 24 to 98 years, with a mean of 63.2 years.

Jaundice was present in 49 patients (24.9%), fever exceeding 38° C was present in 25 (12.7%), and Charcot's triad was present in 38 (19.3%). Seven patients (3.6%) showed progression of biliary sepsis (severe acute cholangitis[5]): mental confusion and shock in one patient; shock and disseminated intravascular coagulopathy (DIC) in one; shock in 4; and DIC in one.

The serum levels (mean $\pm$ SD) of GOT, GPT, bilirubin amylase, and the white blood cell count were $319 \pm 375$, $255 \pm 208$, $3.4 \pm 2.5$, and $382 \pm 733$ (Caraway units, normal $<135$), and $10.700 \pm 5100$ (count/$mm^3$), respectively. Fifty-five patients (28%) showed serum GOT levels of over 400 Karmen units, and ten (5.1%) over 1,000. However, the magnitude of the elevated serum transaminase did not correlate with the severity of the symptoms (Fig. 2.) Twenty patients (10.2%) had concomitant elevation of serum amylase of over 1,000 Caraway units and were also diagnosed as having biliary pancreatitis.

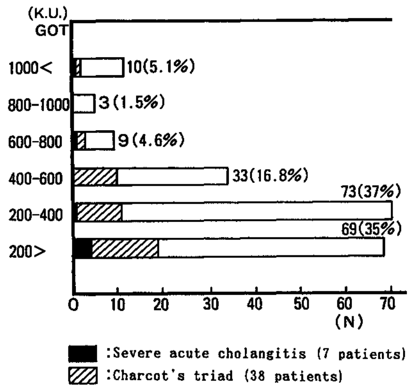

**Figure 2** Magnitude of serum transaminase levels and severity of symptoms. Fifty-five patients (28%) showed serum glutamic oxalacetic transaminase (GOT) levels of over 400, and ten (5.1%) showed levels of over 1.000. The magnitude of the elevated serum transaminase levels, however, did not correlate with the severity of symptoms.

*Preoperative Examinations*

All patients underwent US scan on admission, which revealed gallstones, usually in the swollen gallbladder and/or in the dilated bile duct. Later, some patients were further studied by drip infusion cholangiography, endoscopic retrograde cholangiography (ERC), percutaneous transhepatic cholangiography (PTC), or computed tomography, as indicated in order to detect bile duct stones. The relationship between the imaging studies and the detection of bile duct stones is shown in Table 2. Using these studies, 128 patients (65.0%) were preoperatively diagnosed as having bile duct stones.

*Management*

The management of the 197 patients included: (1) emergency operation within 48 hours of admission in 138 (70.1%, Group I); (2) successful emergency biliary drainage, including PTBD (15 cases) or endoscopic sphincterotomy (ES, 5 cases), in 20 (10.1%, Group II); and (3) initial medical treatment in 39 (19.8%, Group III).

The majority of Group I patients showed no objective improvement in response to conventional supportive therapy such as antibiotic, analgesic, or fluid therapy. ES was unsuccessful in two cases. Deterioration of their general condition and peritonitis were the important factors determining emergency surgery. In some cases, the attending physician performed emergency surgery for the proven SBDS without initial conservative management.

Group II patients underwent PTC or ERC prior to PTBD/ES, which revealed bile duct stones. In nine patients, pus was identified within the bile duct at PTBD/ES. Group III patients responded promptly to initial medical management.

**Table 2** Imaging studies and bile duct stones

| *Imaging Studies* | *Number of cases* | *Detection of BDS** |
|---|---|---|
| (1) US2* | 61 | 29 (47.5%) |
| (2) US + DIC3* | 31 | 14 (45.2%) |
| (3) US + ERC4* | 58 | 49 (84.5%) |
| (4) US + PTC5* | 25 | 23 (92.0%) |
| (5) US + Others | 22 | 13 (65.0%) |
| Total | 197 | 128 (65.0%) |

* : Bile duct stones
2* : Ultrasonography
3* : Drip infusion cholangiography
4* : Endoscopic retrograde cholangiography
5* : Percutaneous transhepatic cholangiogaphy

Group III patients and all Group II patients except one (total of 58 patients) subsequently underwent operation during the same period of hospitalization, after an average of 14 days. In one Group II patient who had previously undergone cholecystectomy, bile duct stones were removed by ES and delayed surgery was not required.

In the seven patients with severe acute cholangitis, emergency surgery was performed in one (Group I), PTBD in five (Group II), and initial medical management in one (Group III).

*Biliary Tract Pathology*

Biliary tract pathology found at emergency surgery (Group I, 138 patients) and at delayed surgery (Group II and Group III, 58 patients) is shown in Table 3. Acute suppurative cholangitis (ASC) is defined as the presence of pus within the bile duct[1]. At emergency surgery, SBDS and ASC were confirmed in 122 (88.4%) and 58 patients (42.0%), respectively. As stated previously, 20 (100%) and nine (45%) of Group II patients had SBDS and ASC, respectively, at emergency PTBD/ES. Accordingly, in the acute stage of the disease, SBDS and ASC were confirmed in 142 (89.9%) and 67 patients (42.4%), respectively, out of a total of 158 patients (138 Group I and 20 Group II patients). Almost all of the patients had acute inflammation of the gallbladder. In the majority, however, the intensity of the inflammation was slight.

Laboratory data on admission and features of the gallbladder stones in the 16 Group I patients and the 18 Group III patients who had no bile duct stones identified at emergency and delayed surgery, respectively, are shown in Table 4. In the 16 Group I patients, four (25%) had biliary pancreatitis and nine (56.3%) had multiple small stones less than 4 mm in diameter. In the 18 Group II patients, four (22.2%) had biliary pancreatitis. Four patients (22.2%) had undergone ES, by which means the bile duct stones had been removed, and no bile duct stone was identified at delayed surgery. The other seven patients (38.9%) had multiple small stones.

*Operative Procedures*

Operative procedures performed at emergency surgery (138 patients) included cholecystectomy alone (cholecystectomy) in five (3.6%), cholecystectomy + choledochotomy or choledocholithotomy (ductal exploration) in 99 (71.7%), and cholecystectomy + choledochotomy or choledocholithotomy + transduodenal

**Table 3** Biliary tract pathology at surgery

| *Biliary tract pathology* | *Emergency surgery (138 cases)* | *Delayed surgery (58 cases)* |
|---|---|---|
| (1) Bile duct stones | 122 (88.4%) | 40 (69.0%) |
| (2) Acute suppurative cholangitis* | 58 (42.0%) | 5 (8.6%) |
| (3) Acute inflammation of the GB2* | | |
| 1) non ~ serous or chronic | 112 (81.2%) | 51 (87.9%) |
| 2) phlegmonous ~ gangrenous | 23 (16.7%) | 7 (12.1%) |
| 3) perforation or abscess | 3 (2.2%) | 0 |

* : Presence of pus within the bile duct
2* : Gallbladder

**Table 4** Laboratory data on admission and features of the gallbladder stones of the 34 patients without bile duct stones

| *No. (Age/Sex)* | *Laboratory data** | | | | *Features of GB stones* | |
|---|---|---|---|---|---|---|
| | *GOT* | *GPT* | *T.bil* | *Amylase* | *Stone Size2** | *Stone Number* |
| Emerg. Surg.3*: | | | | | | |
| 1. (40/♂) | 692 | 945 | 8.7 | 3170 5* | 2 mm | 1 |
| 2. (36/♀) | 299 | 260 | 0.9 | 2521 5* | 4 mm | 3 |
| 3. (68/♂) | 229 | 88 | 1.1 | 2213 5* | 20 mm | 1 |
| 4. (74/♀) | 55 | 37 | 1.1 | 2114 5* | 5 mm | 2 |
| 5. (64/♂) | 210 | 269 | 2.9 | 133 | 2 mm | 486 |
| 6. (54/♂) | 410 | 517 | 8.4 | ? | 4 mm | 200 |
| 7. (50/♂) | 674 | 735 | 2.3 | 132 | 2 mm | 63 |
| 8. (38/♂) | 214 | 134 | 1.0 | 66 | 2 mm | 47 |
| 9. (69/♂) | 716 | 252 | 2.1 | 42 | 3 mm | 31 |
| 10.(42/♂) | 206 | 581 | 2.1 | ? | 3 mm | 28 |
| 11. (62/♂) | 282 | 263 | 3.3 | ? | 4 mm | 12 |
| 12. (73/♀) | 1420 | 816 | 3.9 | 17 | 3 mm | 5 |
| 13. (40/♂) | 636 | 451 | 2.5 | ? | 4 mm | 2 |
| 14. (71/♂) | 332 | 361 | 8.3 | 808 | 20 mm | 2 |
| 15. (60/♀) | 379 | 137 | 2.4 | 747 | ? | 1 |
| 16. (75/♂) | 163 | 263 | 7.6 | ? | 5 mm | 1 |
| Delay. Surg.4*: | | | | | | |
| 1. (65/♀) | 76 | 53 | 0.9 | 4030 5* | 10 mm | 4 |
| 2. (72/♀) | 84 | 35 | 0.6 | 4136 5* | 3 mm | 50 |
| 3. (54/♂) | 197 | 57 | 1.9 | 1929 5* | 6 mm | 1 |
| 4. (26/♀) | 75 | 142 | 1.5 | 1249 5* | 3 mm | 14 |
| 5. (62/♂)6* | 177 | 192 | 3.0 | ? | 1 mm | 4 |
| 6. (73/♂)6* | 89 | 145 | 2.6 | 57 | 10 mm | 5 |
| 7. (36/♂)6* | 173 | 397 | 7.7 | 38 | 13 mm | 1 |
| 8. (36/♂)6* | 153 | 355 | 7.3 | 89 | 16 mm | 1 |
| 9. (44/♀) | 165 | 140 | 1.2 | 111 | 2 mm | 356 |
| 10. (50/♀) | 164 | 144 | 1.0 | 300 | 4 mm | 137 |
| 11. (63/♀) | 579 | 605 | 3.6 | ? | 4 mm | 82 |
| 12. (63/♀) | 252 | 417 | 1.1 | 37 | 6 mm | 44 |
| 13. (75/♂) | 292 | 284 | 3.0 | ? | 1 mm | 30 |
| 14. (52/♂) | 173 | 345 | 13.1 | 427 | 3 mm | 18 |
| 15. (84/♂) | 157 | 74 | 3.3 | ? | 3 mm | 6 |
| 16. (84/♀) | 79 | 101 | 1.4 | 482 | 4 mm | 4 |
| 17. (52/♀) | 104 | 174 | 1.0 | 352 | 15 mm | 4 |
| 18. (81/♀) | 80 | 46 | 1.1 | 33 | 8 mm | 2 |

* GOT; serum glutamic oxalacetic transaminase (Karmen unit, N < 40), GPT; serum glutamic pyruvic transaminase (Karmen unit, N < 35), T. bil; total bilirubin (mg/dl, N < 1.2), Amylase (Caraway unit, N < 135)
2* Minimum size of gallbladder stones
3* Emergency surgery
4* Delayed surgery
5* Biliary pancreatitis (Amylase activity > 1,000)
6* Patients undergoing laparoscopic cholecystectomy after endoscopic sphincterotomy (ES)

papilloplasty or choledochoduodenostomy (drainage procedure) in 34 (24.7%). At delayed surgery (58 patients), cholecystectomy, ductal exploration, and drainage procedure were performed in seven (12.1%), 33 (56.9%), and 13 patients (22.4%), respectively. The remaining five (8.6%) underwent laparoscopic cholecystectomy, among whom four underwent laparoscopic cholecystectomy after ES.

### *Outcome*

Two patients in Group I died; the mortality rate was thus 1.0% (2/197). These two patients were a 66-year-old male and a 79-year-old male who both had SBDS and underwent ductal exploration. The former died of ruptured hepatic artery aneurysm, and the latter of aspiration pneumonia, 13 days and 12 days, respectively, after the initial biliary surgery. The postoperative courses in the remaining 195 patients were generally uneventful. There were no deaths associated with severe acute cholangitis.

### DISCUSSION

Several attempts have been made to define the clinical and laboratory criteria that would identify high-risk groups of patients with choledocholithiasis[6–10], SBDS[11], or acute cholangitis[2,12]. Liver function tests have been reported to be useful for identifying patients with choledocholithiasis[7,9], SBDS[11], and acute cholangitis[2]. Few studies, however, have specifically explained the reasons why liver function tests were useful. In the study by Saharia *et al.*,[2] elevated serum transaminase levels were considered to be a reflection of the pathological condition of the bile duct[2].

We have adopted the clinical concepts of gallstone hepatitis as clinical and laboratory criteria for identifying high-risk patients with acute cholangitis or SBDS. The reasons were: (1) gallstone hepatitis might be a sign of early biliary obstruction caused by impacted bile duct stones; and (2) SBDS might result from biliary tract obstruction, and acute cholangitis from biliary tract obstruction and subsequent infection. As a result, 197 patients with acute gallstone disease and concomitant elevation of serum transaminases (gallstone hepatitis) were suspected of having acute cholangitis or SBDS. Among them, 158 (80.2%) underwent emergency treatment either surgery (138 patients) or PTBD/ES (20 patients). In the acute stage of the disease, the majority (142; 89.9%) of the 158 patients were confirmed to have SBDS, and less than half (67; 42.4%) were confirmed to have ASC.

The majority of patients who had no bile duct stones identified at surgery had either biliary pancreatitis or multiple small stones in the gallbladder. Biliary pancreatitis is thought to be caused by the migration of a stone into or through the ampulla of Vater[13]. Accordingly, a considerable proportion of those patients may have either passed stones or small stones not detected by operative cholangiograms[8,14].

The majority of patients had also acute inflammation of the gallbladder. The intensity of the gallbladder inflammation, however, was slight. Accordingly, acute inflammation of the gallbladder in patients with gallstone hepatitis was considered to be secondary to SBDS[4].

Seven patients had severe acute cholangitis. Our previous study demonstrated necrosis of liver cells and cholangitis in liver biopsy specimens taken from patients with gallstone hepatitis[3,4]. It must be stressed that elevation of serum transaminases in patients with gallstone hepatitis is a reflection of liver cell necrosis caused by impacted bile duct stones. Once the bile duct is obstructed by impacted bile duct stones, it becomes a closed system filled with bile, and pathological changes in the bile duct such as bile statis, increased pressure in the biliary tract or infection may affect the liver cells which bound the bile canaliculus and might cause hepatocellular necrosis[3,4]. Thus, it is possible that bacterial cholangiovenous reflux might occur through damaged tight junctional complexes and also directly through hepatocytes[15] if bile stasis and infection remain in the bile duct, leading to the development of severe acute cholangitis as shown in the case presented. The operative mortality rate in the 196 patients of whom 63 (32.4%) and seven (3.6%) had ASC and severe acute cholangitis, respectively, was 1.0% (2/196). This low mortality rate could be attributable to: (1) early biochemical prediction of SBDS and acute cholangitis by gallstone hepatitis; and (2) initial non-operative management for the majority of patients with severe acute cholangitis using PTBD/ES.

In conclusion, the results of our study show that gallstone hepatitis correctly predicted SBDS and acute cholangitis in 89.8% and 42.4% of attacks, respectively. In patients with acute gallstone disease, gallstone hepatitis indicates that SBDS and acute cholangitis are probable, and facilitates rapid selection of patients for urgent biliary tract exploration. Patients with gallstone hepatitis could develop into severe acute cholangitis and should be managed promptly and properly.

### REFERENCES

1. Boey, J. H. and Way, L. W. (1980) Acute Cholangitis. *Ann. Surg.*, **191**, 264–270.
2. Saharia, P. C. and Cameron, J. L. (1976) Clinical management of acute cholangitis. *Surg. Gynecol. Obst.*, **142**, 369–372.
3. Isogai, M. (1985) A clinicopathological study on the pathogenesis of hepatitis caused by gallstone (in Japanese). *Jpn. J. Gastroenterol. Surg.*, **18**, 1650–1658.
4. Isogai, M., Hachisuka, K., Yamaguchi, A. and Nakano, S. (1991) Etiology and pathogenesis of marked elevation of serum transaminase in patients with acute gallstone disease. *HPB Surgery*, **4**, 95–107.
5. Lay, E. C. S., Tam, P., Paterson, I. A., Ng, M. M. T., Fan, S. T., Choi, T. K. and Wong, J. (1990) Emergency surgery for severe acute cholangitis: The high-risk patients. *Ann. Surg.*, **211**, 55–59.
6. Reiss, R., Deutsch, A. A., Nudelman, I., Kott, I. and Tiqua, P. (1984) Statistical value of various clinical parameters in predicting the presence of choledochal stones. *Surg. Gynecol. Obst.*, **159**, 273–276.
7. Santo, P. D., Kazarian, K. K., Rogers, J. F., Bevins, P. A. and Hall, J. R. (1985)Prediction of operative cholangiography in patients undergoing elective cholecystectomy with routine liver function chemistries. *Surgery*, **98**, 7–11.
8. Taylor, T. V., Armstrong, C. P., Rimmer, S., Lucas, S. B., Jeacock, J. and Gunn, A. A. (1988) Prediction of choledocholithiasis using a pocket microcomputer. *Br. J. Surg.*, **75**, 138–140.
9. Metcalf, A. M., Ephgrave, F. K. S., Dean, T. R., PA-C. and Maher, J. W. (1992) Preoperative screening with ultrasonography for laparoscopic cholecystectomy: An alternative to routine intraoperative cholangiography. *Surgery*, **112**, 813–817.
10. Joyce, W. P., Keane, R., Burke, G. J., Daly, M., Drumm, J., Egan, T. J. and Delaney, P. V. (1991) Identification of bile duct stones in patients undergoing laparoscopic cholecystectomy. *Br. J. Surg.*, **78**, 1174–1176.
11. Anciaux, M. L., Pelletier, G., Attali, P., Meduri, B., Liguory, C. and Etienne, J. P. (1986) Prospective study of clinical and biochemical features of symptomatic choledocholithiasis. *Dig. Dis. Sci.*, **31**, 449–453.
12. Gigot, J. F., Leese, T., Dereme, T., Couninho, J., Castaing, D. and Bismuth H. (1989) Acute cholangitis. Multivariate analysis of risk factors. *Ann. Surg.*, **209**, 435–438.
13. Howard, J. M. (1987) Gallstone pancreatitis. In: Howard JM, Jordan GL, Reber HA (eds) Surgical diseases of the pancreas. *Lea & Febiger, Philadelphia, pp 265*–283.
14. Mayer, A. D. and McMahon, M. J. (1985) Biochemical identification of patients with gallstones associated with acute pancreatitis on the day of admission to hospital. *Ann. Surg.*, **201**, 68–75.
15. Raper, S. E., Barker, M. E., Jones, A. L. and Way, L. W. (1989) Anatomic correlates of bacterial cholangiovenous reflux. *Surgery*, **105**, 352–359.

